# Anticipated HIV stigma among HIV negative men who have sex with men in China: a cross-sectional study

**DOI:** 10.1186/s12879-020-4778-5

**Published:** 2020-01-15

**Authors:** Chuncheng Liu, Ye Zhang, Stephen W. Pan, Bolin Cao, Jason J. Ong, Hongyun Fu, Dan Wu, Rong Fu, Chongyi Wei, Joseph D. Tucker, Weiming Tang

**Affiliations:** 1 University of California San Diego, La Jolla, CA USA; 2 University of North Carolina Project-China, Guangzhou, China; 3 Kirby Institution, UNSW, Sydney, Australia; 4 Xi’an Jiaotong-Liverpool University, Suzhou, China; 5 Shenzhen University, Shenzhen, China; 6 London School of Hygiene and Tropical Medicine, London, UK; 7 Eastern Virginia Medical School, Norfolk, VA USA; 8 Guangzhou CDC, Guangzhou, China; 9 Rutgers – The State University of New Jersey, New Brunswick, NJ USA; 10 Dermatology Hospital of Southern Medical University and the University of North Carolina Project-China, No.2 Lujing Road, Guangzhou, 510095 China; 11 University of North Carolina Chapel Hill, Chapel Hill, NC USA; 12 Institute of Global Health and STI Research, Southern Medical University, Guangzhou, China

**Keywords:** Anticipated HIV stigma, Men who have sex with men, HIV self-testing, Online sex-seeking, disclosure

## Abstract

**Background:**

Anticipated HIV stigma, i.e., the expectation of adverse experiences from one’s seroconversion, is associated with both negative psychological and behavioral outcomes. We know little about anticipated HIV stigma’s relationship with emerging technologies, such as HIV self-testing (HIVST) and online sex-seeking platforms, that have become popular among populations that are disproportionately affected by HIV/AIDS. This study examined correlates of anticipated HIV stigma among Chinese men who have sex with men (MSM).

**Methods:**

In July 2016, MSM, who were ≥ 16 years old and self-reported as HIV negative or unknown, were recruited from a gay mobile phone application in China. Information regarding socio-demographics, sexual behaviors, sexual health service utilization, and anticipated HIV stigma were collected. Anticipated HIV stigma (i.e., negative attitude toward future stigmatization of HIV seroconversion by others) was measured as the mean score from a 7-item Likert-scale ranging from 1 (low) to 4 (high). Generalized linear models were conducted to examine the factors associated with the anticipated HIV stigma scores.

**Results:**

Overall, 2006 men completed the survey. Most men completed high school (1308/2006, 65.2%) and had an annual personal income of ≤9200 USD (1431/2006, 71.3%). The mean anticipated HIV stigma score for the participants was 2.98 ± 0.64. Using social media to seek sexual partners was associated with higher anticipated HIV stigma (Adjusted β = 0.11, 95% confidence interval (CI): 0.05 to 0.17, *p* = 0.001). HIV self-testing (Adjusted β = − 0.07, 95%CI: − 0.13 to − 0.01, *p* = 0.02) and having disclosed one’s sexual orientation to a healthcare provider (Adjusted β = − 0.16, 95%CI: − 0.22 to − 0.96, *p* < 0.001) were associated with lower anticipated HIV stigma.

**Conclusion:**

Our data suggested that anticipated HIV stigma is still common among Chinese MSM not living with HIV. Tailored anti-HIV stigma campaigns on social media are especially needed, and the promotion of HIVST may be a promising approach.

## Background

HIV-related stigma refers to a social process of devaluation that reinforces negative thoughts about people living with HIV (PLHIV) [1]. Abundant evidence shows that men who have sex with men (MSM) experience higher levels of HIV-related stigma compared with other social groups, especially in low and middle-income countries (LMIC) [2]. Studies indicate that this high level of HIV-related stigma is particularly due to MSM’s increased risk of HIV infection and public cultural discrimination of their sexual orientation [2–4].

Most current literature of MSM and HIV-related stigma focuses on the stigma’s influence on MSM living with HIV (MSMLHIV), which suggests that HIV-related stigma may increase adverse mental and physical health outcomes [3–5]. Additionally, HIV-related stigma may contribute to MSMLHIV’s poor retention in healthcare services and antiretroviral adherence [6, 7]. Globally, stigma experiences of MSMLHIV are positively associated with their poorer HIV knowledge, fears toward HIV incurability, and a higher likelihood of high-risk sexual behaviors [8, 9], which could be effectively mitigated by supports from MSMLHIV groups and MSM community-based organizations [10, 11]. Scholars also note that HIV stigma is not only about MSMLHIV individuals, but the social environment they live in. Therefore, many interventions are developed to decrease the HIV stigma through improving the general population’s knowledge about HIV and reducing their prejudice towards MSMLHIV [12].

A growing number of studies have demonstrated that HIV stigma also influences HIV negative individuals through anticipation. Anticipated HIV stigma is the expectation of an individual that they would experience prejudice, rejection, and bias if they became HIV infected. It is an influential factor in high-risk sexual behaviors and poorer health service utilization [13]. For example, scholars have shown that the expectation of future stigmatization from others may act as barriers for HIV negative individuals to find help from health professionals [14–17]. Literature also indicates that people with higher anticipated stigma might have less social support, lower trust in health care providers, and is linked with negative mental health status [13, 18]. Since anticipated HIV stigma is a key barrier to HIV prevention and general health outcome among MSM, there is an urgent call for approaches to decrease the anticipated HIV stigma, an area we know little about [19]. Our study tries to understand the situation of anticipated HIV stigma and investigate its potential correlates, which are essential for designing an effective health campaign for MSM.

Emerging technologies that have been widely used by MSM may offer some challenges and opportunities for anticipated HIV stigma control. For example, HIV self-testing (HIVST) as an alternative approach to HIV testing is increasingly being used by MSM. It is potentially considering an effective strategy to normalize HIV testing in low and middle-income counties (LMIC) [20]. There is wide qualitative evidence that HIVST has removed the test-associated stigma, increased access to treatment, and encouraged the normalization of HIV, especially among the key populations [21, 22]. Likewise, previous research has shown that gay social media platforms have the potential ability to influence MSM attitudes toward HIV infection and have dramatically changed health care utilization and sex-seeking behaviors of MSM [23, 24]. However, systematical quantitative evidence is still absent to demonstrate these potential associations.

This study aims to assess anticipated HIV stigma among MSM in China, one of the biggest LMIC in the world. Chinese society is still relatively conservative and stigmatizes HIV and sexual minorities in various ways [5, 25], which urges for more studies and interventions to be developed. We will explore correlations between anticipated HIV stigma and MSM’s sexual behaviors, HIV and sexually transmitted infections (STIs) testings, sexuality disclosures, and community engagements, with a specific focus on the utilization of emerging technologies such as HIVST and social media use for seeking sex partners.

## Methods

### Study participants

Data for this study derives from the baseline, an online survey of a randomized controlled trial (RCT). The aim of the RCT was to investigate the effect of a comprehensive crowdsourced intervention to increase HIV testing uptake among Chinese MSM. A detailed description of the study design was provided in the published protocol [26]. This baseline survey was conducted between June and August 2016 in eight cities in China (Guangzhou, Shenzhen, Zhuhai, Jiangmen, Jinan, Qingdao, Jining, and Yantai). Participants were recruited through banner advertisements on Blued (Blue Brother, Beijing, China), the largest mobile phone application for MSM social networking in China, with over 40 million users worldwide [27]. MSM, who clicked the survey link was directed to the information page and survey, hosted by SoJump (Sojump, Shanghai, China). Before commencing the survey, we obtained online consent from each participant. Eligibility criteria for the study included being born biologically male, age 16-years or older, currently living in one of the eight study cities, never being diagnosed with HIV infection, and ever had oral or anal sex with a man. Participants’ mobile phone numbers were verified by the survey platform to avoid multiple answers by the same participant. After finishing the survey, each participant received 50 Chinese Yuan (approximately 8 USD) as a mobile phone credit.

### Outcome evaluation

Anticipated HIV stigma in this study was assessed through a seven-item questionnaire, developed by Golub and Gamarel [15] for a study among MSM in New York. Survey items included the expectations of discrimination, prejudice from others, and their feelings if they were infected (Table 2). All the items were rated on a four-point Likert scale (1 = Strongly Disagree; 2 = Disagree; 3 = Agree; 4 = Strongly Agree). The mean of the seven-item scale was calculated to create a final anticipated HIV stigma score, which ranged between 1 to 4. A higher score indicated a higher anticipated HIV stigma. Scale reliability was high in this survey (Cronbach’s α = 0.85). The Chinese translation of the questionnaire was piloted with 15 MSM from two study cities, and amendments were made based on their feedback.

### Measures

Sociodemographic information, including age, education level, income, marital status, gender identity, and self-identified sexual orientation, were collected from each eligible participant. We dichotomized the educational level at high school and annual income at 9200 USD as they were the mean educational and income level of urban China [28]. Self-identified sexual orientation was classified as gay or other.

We also collect behavioral information including usage of social media sexual partners seeking in the past 12 months, ever had sex in the previous 3 months, any condomless sex with male sexual partner(s), and having more than one male sexual partner in the previous 3 months. We also assessed social media usage (ever vs n.ever) of both gay apps such as Blued and Grindr, and non-gay apps such as Wechat, QQ (both mobile text and voice messaging communication services in China) and Weibo (a China-based social networking and microblogging service websites, similar to Twitter). Testing and health care utilization behaviors were also reported by participants, including ever tested for HIV, ever self-tested for HIV, ever facility tested for HIV, ever tested for HIV in the past 3 months, and ever utilized any public sexual health service in the previous 12 months. Public sexual health services included: free condoms and lubricant, peer-led sexual education, HIV and STI screening and treatment, pamphlets on HIV/STI-related information, and medical treatment in the public medical facility.

We collected data about levels of gay-community engagement in sexual health and MSM status disclosure of the participants. We used a six-item scale to measure participants, the level of gay-community engagement, and categorized community engagement into four categories, ranging from no engagement to substantial engagement (see the details questions in the appendix) [29]. MSM status disclosure was defined as having discussed sexual orientation or MSM sexual history with a health professional or others.

### Statistical analysis

Descriptive analysis of socio-demographic, sexual behavior, and HIV/STI testing related variables were conducted by reporting distribution frequencies among the survey participants. Bivariable and multivariable generalized linear models were used to assess measures of association between anticipated HIV stigma with sexual behaviors, testing, and health care utilization behaviors, and community engagement variables. Sociodemographic characteristics, including age, education, income, sexual orientation, and marital status, and city, were adjusted in the multivariable generalized linear models. All analyses were conducted in SPSS version 22.0 (Armonk, NY, UCA). In the model, we defined statistical significance as *p* < 0.05.

### Ethics statement

Eligible survey participants were invited to take part in the survey after indicating their informed consent online. The study protocol was approved by the ethics review committees at the Guangdong Provincial Centre for Skin Diseases and STI Control, the University of North Carolina at Chapel Hill, and the University of California, San Francisco.

## Results

### Sociodemographic characteristics

Overall, the survey link was visited by 25,141 unique IP addresses, and 1003 did not complete the surveyor did not sign the informed consent form. In total, 2006 participants met the inclusion criteria and finished the survey. The mean age of the participants was 25.9 ± 6.4 years. The majority of the participants had received at least a high school diploma or its equivalent (1308/2006, 65.2%), annual personal income less than or equal to 9200 USD (1431/2006, 71.3%), were never married (1733/2006, 86.4%), and self-identified as gay (1446/2006, 72.1%) (Table 1).

**Table 1:** Sociodemographic, sexual behavior, and healthcare utilization behaviors among MSM in eight Chinese cities, 2016 (*N* = 2006)

	Total	%	Stigma score (SD^a^)
SOCIODEMOGRAPHICS			
Age (Mean ± SD)	25.9 ± 6.4		
Educational level			
High school or lower	698	34.8	2.85 (0.63)
Higher than high school	1308	65.2	3.04 (0.64)
Annual income (USD)			
≤ 9200	1431	71.3	2.96 (0.65)
> 9200	575	28.7	3.02 (0.63)
Marital status			
Never married	1733	86.4	2.96 (0.65)
Ever married	273	13.6	3.06 (0.61)
Self-identified sexual orientation			
Gay	1446	72.1	2.96 (0.65)
Others^b^	560	27.9	3.02 (0.64)
SEXUAL BEHAVIORS			
Ever used social media for a sexual partner seeking in the past 12 months			
Yes	1454	72.5	3.01 (0.64)
No	552	27.5	2.89 (0.64)
Engaged in condomless sex in the past 3 months			
Yes	552	27.5	3.03 (0.63)
No	1454	72.5	2.99 (0.66)
Have multiple male sexual partners in the past 3 months			
Yes	591	29.5	3.05 (0.65)
No	1415	70.5	2.94 (0.64)
Ever had sex with female			
Yes	462	23.0	3.04 (0.63)
No	1544	77.0	2.96 (0.65)
HEALTHCARE UTILIZATION			
Ever tested for HIV			
Yes	1216	60.6	2.97 (0.65)
No	790	39.4	2.98 (0.64)
Ever self-tested for HIV			
Yes	621	31.0	2.93 (0.66)
No	1385	69.0	3.00 (0.64)
Ever facility-based tested for HIV			
Yes	1041	51.9	2.97 (0.65)
No	965	48.1	2.98 (0.64)
Utilized any governmental sexual health services in the past 12 months			
Yes	1203	60.0	2.93 (0.66)
No	803	40.0	3.04 (0.62)
DISCLOSURE & COMMUNITY ENGAGEMENT IN SEXUAL HEALTH			
Ever disclosure^c^			
Yes	1348	67.2	2.95 (0.65)
No	658	32.8	3.04 (0.63)
Ever disclosure to a health provider			
Yes	548	27.3	2.87 (0.66)
No	1458	72.7	3.01 (0.63)
Community engagement in sexual health			
No engagement	301	15.0	3.06 (0.62)
Minimal engagement	258	12.9	3.04 (0.62)
Moderate engagement	934	46.6	3.01 (0.63)
Substantial engagement	513	25.6	2.84 (0.68)

^a^*SD* Standard Deviation

^b^Others include people who identified him/herself as straight, bisexual, and others

^c^The disclosure was defined as having discussed sexual orientation or sexual history with men to people other than male sex partners

### Anticipated HIV stigma

**Fig. 1:**
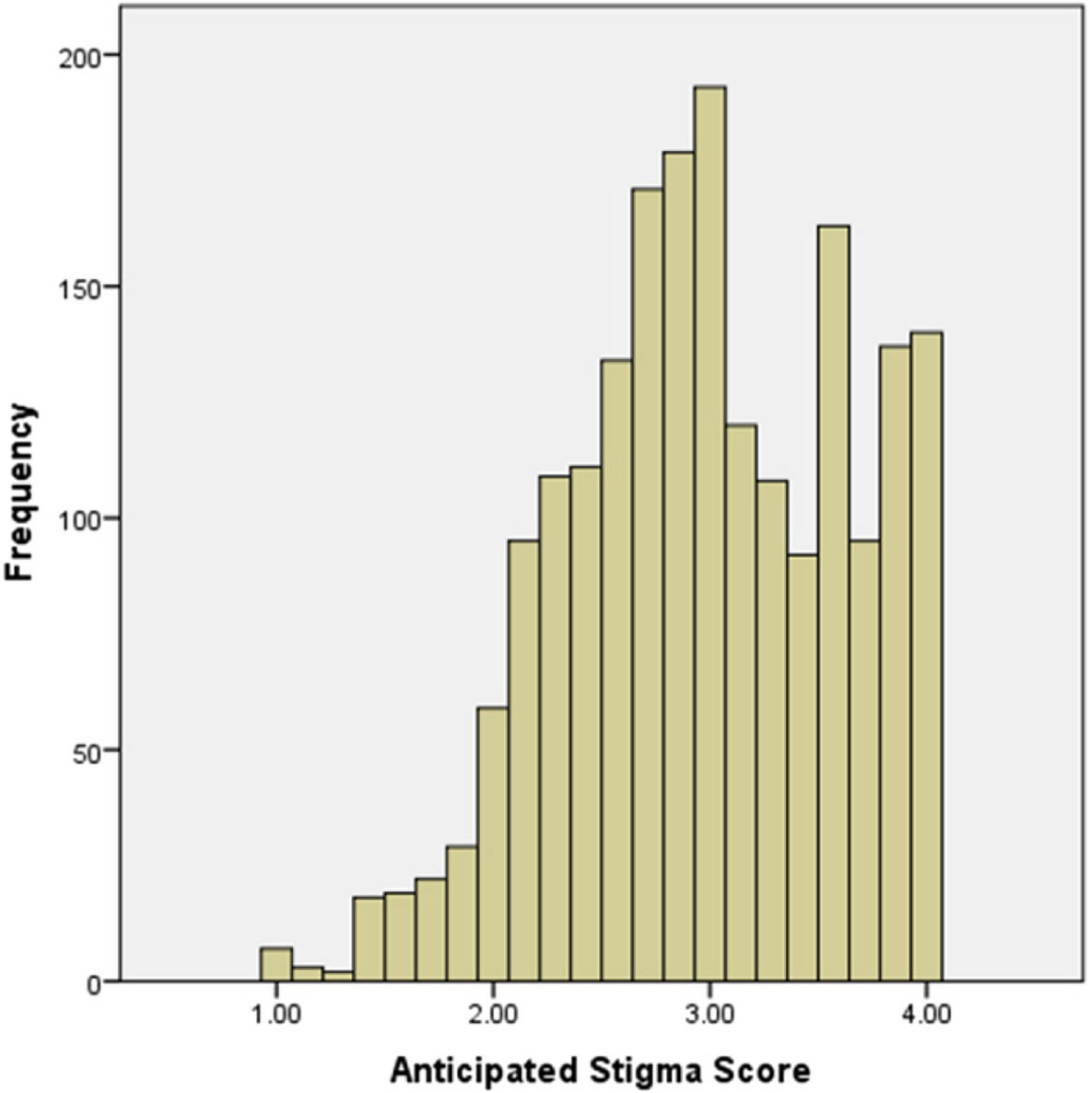
Distribution of anticipated stigma score of MSM in eight Chinese cities (*N* = 2006)

The mean anticipated HIV stigma score among the participants was 2.98 ± 0.64. The distribution of participants’ scores is non-normal (Fig. 1). The strongest anticipated HIV stigma for participants was being discriminated against in general by others (mean = 3.29 ± 0.81), followed by the exception that no one would have sex with him (mean = 3.29 ± 0.80), and no one would date or become involved with him (mean = 3.12 ± 0.86). Participants also showed strong intention to work hard to keep their HIV status as a secret (mean = 2.99 ± 0.92), anticipating isolation from the rest of the world (mean = 2.93 ± 0.93), feeling not as good a person as others (2.70 ± 0.99) and feeling ashamed for having HIV in general (mean = 2.55 ± 0.92) (Table 2).

**Table 2:** Anticipated HIV stigma of MSM in eight Chinese cities, 2016 (*N* = 2006)

Item	Mean (SD)
If I had HIV, I’d worry about people discriminating against me	3.29 (0.81)
If I got infected with HIV, no one would date or become involved with me	3.12 (0.86)
If I got infected with HIV, no one would want to have sex with me	3.26 (0.80)
If I got infected with HIV, I would work hard to keep my HIV status a secret	2.99 (0.92)
If I got infected with HIV, I would feel set apart and isolated from the rest of the world	2.93 (0.93)
If I got infected with HIV, I would feel I was not as good a person as others	2.70 (0.99)
I would feel ashamed of getting HIV	2.55 (0.92)
Overall^a^	2.98 (0.64)

*SD* standard deviation

^a^Cronbach’s α = 0.85

### Sexual behaviors, healthcare utilization, and community engagement in sexual health

Most men have used social media to find sexual partners in the past 12 months (1454/2006, 72.5%), had sex with men in the past 3 months (1165/2006, 58.1%), did not have multiple male sexual partners in the past 3 months (1415/2006, 77%), and never had sex with a female (1544/2006, 77.0%). Among those who had sex with a male in the past 3 months, half of them had engaged in condomless anal sex (552/1165, 47.4%). Around three-fifths of the participants have ever used HIV testing (1216/2006, 60.6%), and around half of the participants ever tested in a facility-based testing site (1041/2006, 51.9%). However, the majority of the participants did not test for HIV in the past 3 months (1382/2006, 68.9%) and never self-tested (1385/2006, 69.0%). Around three-fifths (1203/2006, 60.0%) of the participants have utilized some public sexual health services in the past 12 months. In addition, around two-thirds of the participants ever disclosed (1348/2006, 67.2%) their sexual orientation to others, but seldom disclosed to a health provider (548/2006, 27.3%). Regarding community engagement in sexual health, a few participants have never engaged (301/2006, 15.0%) or engaged at a minimal level (258/2006, 12.9%). About half of the participants had moderate levels of community engagement (934/2006, 46.6%), and a quarter had a substantial level of community engagement (513/2006, 25.6%) (Table 1).

### Association between anticipated HIV stigma and behavioral variables

Adjusting for demographic background, participants who used social media for male sexual partner seeking in the past 12 months (Adjusted β = 0.11, 95% CI: 0.05 to 0.17), ever had condomless sex in the past 3 months (Adjusted β = 0.07, 95%CI: 0.01 to 0.13) and had multiple male partners in the last 3 months had a higher level of anticipated stigma (Adjusted β = 0.10, 95%CI: 0.04 to 0.16), compared with their respective comparison groups (Table 3).

**Table 3:** Bivariable and multivariable generalized linear models of anticipated HIV stigma and behavioral variables among MSM in eight Chinese cities, 2016 (N = 2006)

	Unadjusted β (95%CI)	p	Adjusted β (95% CI)^a^	p
SEXUAL BEHAVIORS				
Using social media for sexual partner seeking in the past 12 months	0.12 (0.05, 0.18)	< 0.001	0.11 (0.05, 0.17)	0.001
Ever had condomless sex in the past 3months	0.03 (−0.04, 0.11)	0.40	0.07 (0.01, 0.13)	0.03
More than one male sexual partner in the past 3 months	0.11 (0.05, 0.17)	< 0.001	0.10 (0.04 to 0.16)	0.002
HEALTHCARE UTILIZATION				
Ever tested for HIV	0.00 (−0.06, 0.05)	0.89	−0.03 (− 0.09, 0.03)	0.34
Ever self-tested for HIV	−0.06 (− 0.12, 0.00)	0.04	− 0.07 (− 0.13, − 0.01)	0.02
Ever facility tested for HIV	−0.01 (− 0.07, 0.05)	0.68	−0.03 (− 0.09, 0.03)	0.29
Utilized any governmental sexual health services in the past 12 months	− 0.11 (− 0.16, − 0.05)	< 0.001	−0.12 (− 0.17, − 0.06)	< 0.001
DISCLOSURE & COMMUNITY ENGAGEMENT IN SEXUAL HEALTH				
Ever disclosure^b^	− 0.09 (− 0.15, − 0.03)	0.003	−0.09 (− 0.15, − 0.03)	0.004
Ever disclosed to health provider	−0.14 (− 0.20, − 0.08)	< 0.001	−0.16 (− 0.22, − 0.09)	< 0.001
Being substantially engaged in the community in sexual health^c^	−0.22 (− 0.32, − 0.13)	< 0.001	−0.23 (− 0.32, − 0.14)	< 0.001

^a^The multivariable analysis controlled for age, education, income, sexual orientation, marital status, and city

^b^The disclosure was defined as having discussed sexual orientation or sexual history with men to people other than male sex partners

^c^Referent: no engagement

Participants who had ever self-tested for HIV (Adjusted β = − 0.07, 95%CI: − 0.13 to − 0.01) had a lower level of anticipated HIV stigma compared with their counterparts. Those who utilized public sexual health services in the past 12 months also had a lower level of HIV anticipated stigma (Adjusted β = − 0.12, 95%CI: − 0.17 to − 0.06) (Table 3).

Participants who had ever disclosed their sexuality or sexual history to people other than their partner (Adjusted β = − 0.09, 95%CI: − 0.15 to − 0.03), or disclosed to a health provider (Adjusted β = − 0.16, 95%CI: − 0.22 to − 0.09) had lower anticipated HIV stigma. Men who have substantial community engagement in sexual health issues (Adjusted β = − 0.23, 95%CI: − 0.32 to − 0.14) had a lower level of anticipated HIV stigma score compared with those who had never engaged (Table 3).

## Discussion

Anticipated HIV stigma is associated with many adverse outcomes among MSM [14–16]. Our study contributes to the literature by examining the correlates of anticipated HIV stigma in China and exploring the relationship between stigma and emerging technologies (i.e., HIVST and social media use). We found that anticipated HIV stigma was negatively associated with HIVST and disclosure to the health provider, while positively associated with using social media for sex seeking.

We found that the overall anticipated HIV stigma is still high among Chinese MSM. This finding is consistent with previous studies using different scales and measurements [14, 27, 30]. The level of anticipated stigma we found is higher than that reported among MSM in the United States (2.98 versus 2.57) [15], which could be explained by differences in culture and societal backgrounds between these two countries. Here we would highlight two potential reasons for higher anticipated HIV stigma among Chinese MSM. First, HIV is still severely and commonly believed to be associated with moral deficiency and deviant behaviors in China [30, 31]. This is compounded by HIV prevention materials, which may inadvertently increase such stigma through images that seek to frighten people into healthy behaviors [27, 30, 32]. Second, sex education (including accurate information regarding HIV transmission) is relatively lacking in China. Inaccurate beliefs may generate unnecessary fears towards HIV/AIDS and exaggerated bad life experiences in people’s anticipations. This exaggeration effect is especially salient among MSM, as they are commonly depicted as an HIV high-risk population. As a result, they commonly worry about HIV/AIDS more compared with the general population [30, 32].

Our results suggested that HIVST history was associated with less anticipated HIV-related stigma. This is consistent with existing literature showing that general HIV testing history is associated with lower HIV-related stigma [16, 18, 31, 33]. However, our focus on HIVST is novel. Previous studies indicated that HIVST could ensure greater privacy compared to facility HIV testing [20–22]. Thus, HIVST may reduce MSM’s fears toward HIV testing because they could worry less about stigmatized experiences that are associated with unintended status disclosure. Meanwhile, HIVST is relatively easy to conduct without a psychological burden compared with the facility HIV testing [22]. Admittedly, social and ethical considerations have been raised by scholars, such as the potential for coercion and adverse emotional impacts [34]. Yet a recent systematic review showed that HIVST, in general, would empower people and is the means by which many MSM have their first HIV test [22]. Utilization of HIVST provides an opportunity for MSM to attain better knowledge and increase their awareness about HIV [35], which may enable them to better understand the testing process and mitigate their anxiety and fear of HIV infection.

We found that men who used social media for sex seeking had higher anticipated HIV stigma. This is consistent with a Nigerian study showing that online sex-seeking was associated with the higher stigma of same-sex behavior [36]. A few reasons might explain the connection between anticipated HIV stigma and sex-seeking on social media. First, social media allows individuals to have the ability to create and distribute information, which may facilitate the distribution of inaccurate knowledge regarding HIV that could exacerbate stigma [24]. Meanwhile, studies in China and the United States showed that MSM were more likely to have greater perceived HIV-related stigma than the general population because they are more likely to know people living with HIV in their community and observe how they are negatively treated by others [18, 25, 37]. These “anticipated HIV stigma experiences” and discourses may be intensified in online platforms, where there is a higher density of MSM with more opportunities to discuss HIV with others compared with traditional venues [17, 23, 24]. Studies in the United States also showed that in-group stigma towards HIV is commonly seen on MSM-related social media platforms [38].

We found that sexual orientation disclosure, particularly to healthcare providers, was negatively associated with anticipated HIV stigma. This finding is consistent with previous studies on disclosure and MSM related stigma [39, 40], yet inconsistent with another study from 2009 that reported that disclosure to medical providers would increase HIV-related stigma due to the prejudice regarding HIV among medical providers [27]. This inconsistency may reflect the progress that Chinese medical education has made during the last decade, where many anti-HIV stigma projects have been conducted [41, 42]. In recent years, community-based organizations have increasingly launched gay-friendly medical service programs, so MSM could disclose their sexuality and concerns regarding HIV more easily when facing a gay-friendly medical provider [43]. In response, those medical providers could be a source of social support and would be more likely to offer accurate and targeted HIV knowledge and decrease the fear and stigma towards HIV among MSM [36].

This study has several policy implications. First, more anti-HIV-related-stigma campaigns are needed. As our results showed, different forms of anticipated HIV-related stigma still common among MSM in China. More official or public institutions should be involved in generating resources to promote HIV-related knowledge and distribute MSM friendly and accurate knowledge regarding HIV. Due to the rapid growth of social media platforms, and a dearth of online interventions, tailored interventions on social media platforms are urgently needed. Second, promoting HIVST might be a promising method to decrease HIV-related stigma and increase HIV testing rates among MSM in China. The HIVST kits were available on the Chinese online shopping website since 2014. Although China CDC and community-based organizations (CBOs) started to add effort to improving HIVST and its linkage to care, China has no national guideline for HIVST linkage to care yet. The future effort need to devote to developing policies for the use of HIVST to assure the quality of HIVST and its acceptable links to counseling, care, and treatment [44]. Third, more MSM friendly medical services should be provided to facilitate MSM’s willful disclosure of their MSM status, which in the process may contribute to the reduction of stigma.

As a cross-sectional study, our study has several limitations. First, the study design precludes us from making any casual conclusion, and any results from this study should be interpreted by caution. Second, our questionnaire platform did not allow us to distinguish the number of participants who did not complete the survey from the one who did not sign the informed consent. However, many previous studies have shown that HIVST is an effective tool in encouraging HIV testing among people who have barriers to receive traditional facility HIV testing services and rarely tested for HIV previously. Thus, we think our study provided a hypothesis for future research of HIVST effectiveness on decreasing HIV stigma among the key population. Third, participants were recruited from a gay mobile application, which tends to be used by young and well-educated MSM [45]. However, this method could help us reach those hidden populations that are not reached by traditional recruitment methods. Fourth, social desirability may have influenced participants’ responses on the stigma scale. However, our survey was a computer/smart-phone based survey, which ensured a high level of privacy and anonymity to reduce this possibility. Fifth, although our results showed statistically significant correlates of anticipated HIV stigma, the clinical significance of these results need to be further investigated. More qualitative studies are particularly needed in examining the specific mechanism.

## Conclusions

Despite such limitations, we can conclude that the anticipated HIV stigma is still high among Chinese MSM not living with HIV. The finding of this study highlights the opportunities for conducting health campaigns against HIV stigma among MSM and people around them, especially through online platforms.

## Data Availability

The datasets used and/or analyzed during the current study are available from the corresponding author on reasonable request.

## Abbreviations

AIDS: Acquired Immune deficiency syndrome
CI: Confidence interval
HIV: Human immunodeficiency virus
HIVST: HIV self-testing
LMIC: Low and middle-income countries
MSM: Men who have sex with men
MSMLHIV: Men who have sex with men living with HIV
PLHIV: People living with HIV
SD: Standard deviation
STI: Sexually transmitted infections
